# Why do biomedical researchers learn to program? An exploratory investigation

**DOI:** 10.5195/jmla.2020.819

**Published:** 2020-01-01

**Authors:** Ariel Deardorff

**Affiliations:** Library, University of California, San Francisco, San Francisco, CA, ariel.deardorff@ucsf.edu

## Abstract

**Objective:**

As computer programming becomes increasingly important in the biomedical sciences and more libraries offer programming classes, it is crucial for librarians to understand how researchers use programming in their work. The goal of this study was to understand why biomedical researchers who enrolled in a library-sponsored workshop wanted to learn to program in R and Python.

**Methods:**

Semi-structured in-depth interviews were performed with fourteen researchers registered for beginning R and Python programming workshops at the University of California, San Francisco Library. A thematic analysis approach was used to extract the top reasons that researchers learned to program.

**Results:**

Four major themes emerged from the interviews. Researchers wanted to learn R and Python programming in order to perform their data analysis independently, to be an informed collaborator, to engage with new forms of big data research, and to have more flexibility in the tools that they used for their research.

**Conclusions:**

Librarians designing programming workshops should remember that most researchers are hoping to apply their new skills to a specific research task such as data cleaning, data analysis, and statistics and that language preferences can vary based on research community as well as personal preferences. Understanding the programming goals of researchers will make it easier for librarians to partner effectively and offer services that are critically needed in the biomedical community.

## INTRODUCTION

Biomedical research is growing more computationally intensive [1]. Researchers from the basic and clinical sciences are now working with larger data sets and performing more complicated computational tasks across the research life cycle. Whether they are collecting terabytes of data from genomic sequencers, cleaning large electronic health record data sets, or creating algorithms to model complex cellular relationships, the work of biomedical researchers resembles that of computer scientists more than ever before [1].

As their workflows change, researchers in the sciences have realized that they need new tools and techniques to make their work reproducible. It is no longer possible to simply document a manual data-cleaning-and-analysis workflow; instead, new guidelines recommend that researchers automate their steps with programming scripts and version control [2–4]. Unfortunately, many scientists do not learn to program as part of their graduate training and must seek additional courses and workshops to use these new techniques [5].

Seeing an opportunity to provide crucial training, some academic libraries have begun offering programming courses and workshops. The majority of these courses focus on the programming languages R and Python, two free, open source software environments that enable users to clean, analyze, and visualize data as well as perform more complicated research computing tasks [6]. Librarians at the National Institutes of Health, New York University Langone Health, Stanford University, the University of Arizona, and the University of California, Los Angeles, teach R programming to researchers [7–11], and librarians at Purdue University have integrated R programming into their semester-long research data management course for graduate students [12].

Even libraries without local programming experts have begun offering programming workshops through partnerships with nonprofit organizations like Software Carpentry. Software Carpentry workshops are two-day, hands-on courses that cover the building blocks of reproducible scientific computing: the programming languages R or Python, version control with Git, and scripting in Unix [13]. Libraries interested in hosting a workshop pay a small fee to Software Carpentry to recruit instructors and provide the workshop materials to their local communities; those interested in hosting regular workshops can become sustaining members and pay an annual fee [14]. The University of Oklahoma library became a Software Carpentry member in 2014 as a way of teaching “modern research workflows” [15], and the New England Software Carpentry Library Consortium was formed in 2017 to bring together libraries from across the region so that they could offer programming workshops and train instructors from the library community [16].

The University of California, San Francisco (UCSF) Library became a Software Carpentry member in 2016 and has since offered more than 11 workshops to over 700 biomedical researchers. While demand for the workshops is always high, it is not always clear what specific tasks and goals motivate researchers to attend. This makes it harder to target our content (a necessary step as Software Carpentry provides more material than can possibly be taught in one workshop) and measure the impact of the classes. The goal of the current research was to identify the most common reasons that researchers at UCSF enroll in beginning R and Python programming workshops. This will inform the design and scope of future classes and help librarians understand the role of programming in health sciences research.

## METHODS

This project is part of a larger mixed-methods study on the impact of introductory programming workshops on research workflows. For the larger study, the author interviewed participants before and after participating in a library-led programming workshop to learn about their research workflows and their motivations for learning to program. In this paper, I focus on a subset of the qualitative data from the pre-workshop interviews related to researchers’ motivations for learning to program. The remaining pre-workshop qualitative data as well as qualitative and quantitative data from the post-workshop interviews (describing the impact of the workshop on their workflows) will be reported in a subsequent publication. This study was certified as exempt from review by the UCSF Institutional Review Board.

### Study recruitment

I recruited fourteen participants who registered for a two-day, library-led introductory programming workshop in March 2019. These workshops covered an introduction to Git, Unix, and either R or Python and were open to everyone at the university. Approximately thirty-six registered for the R track, and thirty-six registered for the Python track. The number of research participants was selected based on research indicating that twelve interviews is generally sufficient to gather most major themes [17]. The inclusion criteria specified that participants must be currently involved in research and planning on staying at UCSF for six months (to be able to reach them for follow-up interviews). These criteria left a total of fifty-nine possible participants out of the seventy-two enrolled in the course. I used stratified random sampling to select seven participants who registered for the R workshop and seven who registered for the Python workshop. Of the initial fourteen participants selected, seven did not respond and two declined to participate. These were replaced by an additional nine random participants until fourteen were reached.

### Interviews

In January and February 2019, I performed in-depth semi-structured interviews with participants before they took the programming workshop. I asked about the tools and methods they used, pain points in their workflows (which will be discussed in a subsequent publication), and what participants were hoping to learn in their workshops. The focus of this analysis was drawn primarily from the question: “What do you hope to learn in the workshop?” The interviews (supplementary Appendix A) ranged from twenty to forty-five minutes and were recorded and transcribed using the online tool Rev.com. After transcription, I read through each interview transcript while listening to the audio recordings to ensure the content was faithfully reproduced, addressing any errors (for example, “are” instead of “R”) as I found them. Finally, I redacted names of people and groups and generalized research topics to preserve anonymity.

### Data analysis

I analyzed the data using the applied thematic analysis framework, a methodology inspired by grounded theory, positivism, interpretivism, and phenomenology [18]. Because this was largely an exploratory analysis, I used an inductive approach to read through the transcripts, identify major themes, and create corresponding codes. These codes were then elaborated in the code book and applied using an iterative approach. I performed all coding using the online data analysis tool Dedoose. The code book is available in supplemental Appendix B.

## RESULTS

### Participant demographics

The majority of the participants were postdoctoral researchers (nine of fourteen), followed by three research staff, one graduate student, and one faculty member. These demographics were in line with the typical audience of a UCSF programming workshop. The departmental representation was also similar to a typical workshop, with a larger group from neurology (three of fourteen), developmental and stem cell biology (three of fourteen), and immunology (two of fourteen), and the rest coming from orthopedic surgery, neuroscience, neurological surgery, anatomy, pharmacy, and bioethics. As the workshops were marketed to beginning programmers, thirteen of fourteen described themselves a “novice” or “beginner” programmer, and only one participant considered themselves to be an “intermediate” programmer.

### Overall themes in programming goals

Although specific individual learning goals differed, four major themes stood out as reasons why these biomedical researchers wanted to learn to program: independence in data analysis, programming literacy, new kinds of big data research, and tool flexibility. These themes applied across groups of learners, regardless of whether they registered for the R or Python workshop.

#### Independence in data analysis

Independence arose as a theme several times throughout the interviews. Many researchers spoke of working with collaborators in their labs, often bioinformaticians and statisticians, to analyze their research data and feeling uncomfortable because they did not understand the work being done. Their lack of programming skills made it harder for them to ask the right questions and ensure that the research was being carried out in the way they intended. They hoped that learning to program would allow them to analyze their data independently, without needing to rely on someone else. As one researcher shared:

I’m relying on their interpretation of what’s coming out of the data, to then take it and do more functional studies. I mean, the goal is for [the data] to generate more hypotheses and give more information, but I would also like to be able to actually process the data myself and also come to those conclusions independently…Collaboration is wonderful, but it’s also nice to...I feel like I’m at a disadvantage because I don’t understand enough about what’s going on with the data itself. You know what I mean?

For others, this need for independence was compounded by the fact that a previous programming expert was leaving their lab, requiring them to learn the skills themselves or wait several months to find another expert.

#### Programming literacy

While independence in data analysis was a common thread, not everyone was interested in performing all the work themselves. Some researchers were interested in learning to program so that they could read over code and have a general idea of what was happening, a sort of basic programming literacy. Often this was so that they could understand the work of their collaborators enough to ask intelligent questions and plan research tasks accordingly. One participant shared that they wanted to learn more about programming and computational processes in general to be a better program manager, saying that:

My job doesn’t require that I fully understand the nuances that they’re working with, but I think that I could be a more effective project manager if I did, because it also would help me if I knew how long different steps take. I don’t want to be chasing somebody down saying, “You’re delinquent on a project,” and they’re like, “Well, it’s still running on my computer,” that kind of thing.

This same researcher worried that their colleagues “don’t have the language to communicate with computer scientists in a way that people take seriously,” indicating that the programming workshop could help them bridge this gap. Another researcher said that they did not envision themselves writing programs or coding on a regular basis but wanted “to be able to read the code that was done and just quickly find the part of the code that I needed to look closely at.”

#### New kinds of “big data” research

Researchers’ involvement with big data reflected the increasing size of large data sets in the field over the past ten years. They described needing to analyze comma separated values (CSV) files with millions of patient encounters or combine large genomic data sets with patient questionnaires. These data sets were too large to analyze manually using the tools they were familiar with, such as Excel; instead, researchers needed programming skills to manipulate and analyze them.

One particular experimental approach that came up in five of the interviews was RNA sequencing (RNA-seq) and single cell (sc)RNA-seq. These are relatively new techniques used to analyze RNA molecules to study cellular responses, and the process generates a large amount of data that requires programming skills to analyze or even look at the data [19]. One researcher described RNA-seq as the stage:

where I needed all this programing, because like, this data is just like insane...And this is where I need all the tool[s], like the coding and all this software to analyze this data.

As scRNA-seq has grown in popularity, it has required many teams to expand their programming skills. In fact, many participants in this study were tasked with learning enough coding fundamentals to kickstart this new technique in their lab.

In addition to learning skills to apply to specific techniques like scRNA-seq, some participants shared that they saw the rise of big data in the life sciences and wanted to learn programming so that they would have more career opportunities in data science and bioinformatics. One shared that:

I’ve seen labs switching 50, 60% into bioinformatics…and to be honest it seems to be the next industry that is going to be on the rise in terms of science.

#### Tool flexibility

Even though the majority of the participants considered themselves programming beginners or novices, many already had some familiarity with a programming language like R or Python and were attending the workshop in order to switch languages. Of particular note was that five of the seven researchers enrolled in the Python workshop had previous experience using R, whereas only one of the R attendees knew some Python. The researchers who were switching from R to Python said that Python was more common in their field, and they had been told that it was faster and easier to use. One noted that their collaborators were:

now shifting towards Python because it’s much faster at doing the same things…It seems like since they are really pushing for that, we’re going to do the next project we do with them in Python instead of R.

Many Python attendees wanted to learn both R and Python so that they would have increased flexibility in their work. Noting another popular data analysis tool, one researcher shared that:

I want to have a basic understanding of Python, R, and MATLAB, then if I can search any software online, or any course online, relative to this, then I can make use of it.

Many of the researchers who enrolled in the R workshop were planning on switching their data analysis to R from their current graphical user interface (GUI) or “point and click” software so they would not be limited by the capabilities of a particular tool. One R attendee said that learning R would allow them to stop using a variety of single-use data analysis and visualization tools, saying that if they knew R, they “would just stop using SPSS, Origin Pro, anything, you know. Because I know R is simpler in that case. And to save time, also [it’s] more flexible so I can do whatever I want.” Others shared that they were looking into R because it was a free alternative to Stata, found R more user friendly than SAS, or liked all the packages available on GitHub.

## DISCUSSION

### Recommendations for librarians

The programming goals identified in this study can be helpful for librarians to keep in mind as they plan programming workshops for biomedical researchers. First, these interviews reinforced the fact that while biomedical research is rapidly becoming more computationally intensive, many researchers lack the skills to fully engage in these new areas of research. Most of the researchers interviewed for this study did not receive programming instruction as part of their graduate studies and needed to play catch-up to perform research independently or act as informed collaborators. They were, therefore, motivated to attend programming bootcamps or other forms of instruction that could teach them the material and hands-on skills in a short amount of time.

Unlike other programming bootcamps that are designed to transition learners into roles as software developers or app designers, these researchers were planning on using their new skills to more fully engage with their areas of research. They were not learning to program because they loved computers, but because they needed these skills to do their work. Librarians who are interested in offering workshops might, therefore, focus on teaching practical modules related to data cleaning, data analysis, and statistics that can be applied immediately.

This is an area where the Software Carpentry materials are especially helpful, because they are designed to help researchers apply programming to their work and focus on “good enough” methods rather than the fundamentals of computer science. Librarians with programming expertise might consider going beyond these basic classes to offer advanced workshops targeted at specific research pipelines or programming packages. In addition to introductory programming classes, the UCSF Library now offers more advanced classes like “DNA Variant Analysis with R,” “RNA-Seq Analysis with R,” and “Reading Data from an API with Python” that directly address the needs mentioned in this research [20]. At the same time, librarians might also consider offering workshops for a variety of audiences, including a higher-level approach for researchers who want to obtain a basic level of “programming literacy” as well as hands-on sessions for researchers hoping to apply the material to their work.

Librarians involved in planning workshops should remember that the exact programming language chosen (R or Python in this case) depends on a variety of factors including popularity in a specific community, the availability of a particular package, and personal preference. While R started as a statistical programming language, it is now heavily used in bioinformatics research due to the wide variety of data analysis packages available on the Bioconductor portal [21]. Python, on the other hand, is often seen as a more general-purpose programming language popular for data science applications [22]. That said, both R and Python are used throughout biomedical research.

Based on the participants in this study, it might seem like more R users are turning to Python, but the UCSF Library still sees demand for both languages, each of which has its strengths. There may also be some disciplinary differences in language preference (for example, three of the participants switching from R to Python were from neurology/neurological surgery), but this cohort was too small to draw any conclusions. Sometimes the choice boils down to user interface: while one researcher might prefer R Studio (the R environment), another might find Jupyter (the Python environment) more user-friendly. At UCSF, we advise researchers to learn the language that is most used by their research peers as they will more likely find support and tutorials that are relevant to their needs.

Finally, librarians who are interested in this area might consider developing their own programming skills. Library Carpentry (which merged with Software and Data Carpentry in 2018 and is now part of the organization collectively known as “the Carpentries”) provides curricula for librarians who are interested in learning the basics of programming and data organization [23]. There are also several webinars and tutorials developed for librarians, including the Medical Library Association’s webinar on R programming [24] and Library Juice Academy’s webinar on Python [25]. Even a small amount of programming knowledge can make it easier to talk to researchers about their programming goals and plan workshops accordingly and could lead to librarians using programming to further their own research.

### Limitations

The results of this study are drawn from a relatively small cohort of biomedical researchers at UCSF. While I am confident that the motivating factors are representative of our workshops, they might not reflect the needs of other biomedical researchers or researchers outside of the health sciences. While they had similar demographic characteristics (in terms of roles and departments), it is possible that the recruited researchers who declined to participate or did not respond to the research invitation might have had different goals or expectations than those who did. The analysis and coding for this project was performed solely by me, and a different researcher might have interpreted slightly different themes.

## CONCLUSION

Introductory programming workshops can be an excellent way for academic libraries to provide essential services to their research communities. In the health sciences, libraries have seen an increasing need for researchers to use R and Python in order to work with large data sets, engage in new areas of data-intensive research, and collaborate effectively in the era of team science. I hope that the results of this study will help librarians understand why biomedical researchers learn to program so that they can design workshops and programs that better serve researchers’ needs.

## SUPPLEMENTAL FILES

Appendix A Interview protocols

Appendix B Code book

## 

**Ariel Deardorff**, ariel.deardorff@ucsf.edu, https://orcid.org/0000-0001-8930-6089, Library, University of California, San Francisco, San Francisco, CA
